# Oocyte developmental potential and embryo production before puberty in cattle

**DOI:** 10.1590/1984-3143-AR2024-0069

**Published:** 2024-08-16

**Authors:** Joao Henrique Moreira Viana, Bianca Damiani Marques Silva, Rodrigo Martins de Moura, Luiz Fernando Rodrigues Féres, Ricardo Alamino Figueiredo

**Affiliations:** 1 Embrapa Recursos Genéticos e Biotecnologia, Brasília, DF, Brasil; 2 Universidade de Brasília, Brasília, DF, Brasil; 3 Universidade Edson Antônio Velano, Alfenas, MG, Brasil

**Keywords:** cattle, *in vitro* embryo production, sexual maturity

## Abstract

With the development of *in vitro* technologies, embryos can be produced using oocytes retrieved directly from the ovaries, i.e., regardless of ovulation. This has allowed the use of different animal categories as oocyte donors, including prepubertal cattle. The advantages of using this strategy to shorten the generation interval and accelerate genetic gain over time were soon recognized, and the first offspring generated using oocytes collected from calves were born in the early 1990s. Nevertheless, embryo production from calves and prepubertal heifers remains a challenge. The oocytes collected before puberty present low *in vitro* developmental potential, and the subsequent blastocyst rates are consistently lower than those from pubertal females. The acquisition of developmental competence by the oocytes occurs progressively throughout the prepubertal period, which can be subdivided into an early, intermediate, and late prepubertal (or peripubertal) phases, each characterized by different physiological and endocrine features. Therefore, embryo yield increases with age but will only achieve its maximum after puberty. The most common strategy to improve oocyte developmental potential before puberty is the use of gonadotrophic stimulation prior to oocyte retrieval. The results with superstimulation, however, vary among studies, depending on the source, dose, and length of FSH treatment, as well as the age and breed of the donors. The use of calves and prepubertal heifers as oocyte donors should also consider the possible impacts of the oocyte retrieval technique (LOPU or OPU) and the use of exogenous hormones on their subsequent fertility and productive life.

## Introduction

As occurs with artificial insemination, embryo collection *in vivo* requires heifers to be able to ovulate (either naturally or induced) and support the subsequent steps of fertilization and early embryo development in the uterine tubes and uterus. Therefore, embryos can only be collected *in vivo* from heifers around the time of puberty (Fernandes et al., 2020). Embryo production *in vitro*, on the other hand, can be carried out using oocytes collected directly from the ovaries of calves or prepubertal heifers by surgical (Taneja et al., 2000), laparoscopic (Baldassarre et al., 2018), or ultrasound-guided approaches (Landry et al., 2016). The lack of maturity of the hypothalamic-pituitary-ovary axis, however, results in oocytes with reduced developmental potential *in vitro*, and thus in lower blastocyst and pregnancy rates.

The possibility of producing embryos and offspring from oocytes collected from prepubertal calves was demonstrated in the early 1990s (Armstrong et al., 1992). The demand for the use of calves and prepubertal heifers as oocyte donors, however, has been boosted by the recent development and adoption of genome selection by many animal breeding programs (Jiang et al., 2019). As a result, there is renewed interest in a better understanding of the endocrine and physiological mechanisms underlying the follicular dynamics and the acquisition of developmental competence by the oocyte during the prepubertal period, and the possible technical and pharmacological approaches to improve *in vitro* embryo production (IVEP) efficiency from young donors.

The use of prepubertal heifers is particularly important in tropical countries such as Brazil due to the large use of Zebu breeds, known to be less precocious than Bos taurus breeds (Nogueira, 2004). Over the past two decades, IVEP has become the technique of choice for animal breeding programs in Brazil, the country that leads in the number of embryos transferred worldwide (Viana, 2023). In 2009, for example, embryo technologies accounted for an estimated 21.0% of all Nelore and 71.0% of all Gir calves registered by the Brazilian Association of Zebu Breeders (Viana et al., 2017). These are the most important beef and dairy Zebu breeds raised in Brazil, respectively, and thus the possibility of reducing generation intervals by using prepubertal heifers as oocyte donors could result in a significant acceleration of genetic progress over time, with consequent benefits for the beef and dairy production sectors.

The aim of this review is to discuss the changes in oocyte competence throughout the prepubertal period and strategies to improve blastocyst rates based on gonadotrophic priming before oocyte retrieval, as well as the possible consequences for the subsequent fertility.

## Age, sexual maturity and potential as oocyte donor

The blastocyst production, both considering total number and rates, is known to be lower in prepubertal than in pubertal or adult cattle (reviewed by Currin et al., 2021; Baruselli et al., 2021). This difference, however, is not uniform during the prepubertal period, but rather reflects the endocrine, physiologic, and developmental phases that calves and heifers undergo prior to puberty. Each of these phases, therefore, poses different bottlenecks and challenges for the use of prepubertal cattle as oocyte donors.

Although there is a general consensus on the lower IVEP outcomes from prepubertal cattle, when compared with those from pubertal donors, the magnitude of this difference is still a matter of debate. The inconsistencies among studies may be associated with the substantial variation in the age range of experimental groups defined as “prepubertal”, which included calves as early as 2-3 months old (mo) (Taneja et al., 2000; Baldassarre et al., 2018) or as late as 12 mo or older (Oropeza et al., 2004; Camargo et al., 2005). Moreover, these studies used different donor preparation protocols, oocyte retrieval techniques, and *in vitro* culture systems, as well as different control groups (e.g., pubertal heifers, adult cows, or abattoir ovaries), resulting in a large range in the blastocyst rates reported for prepubertal cattle (e.g., from 0% [Presicce et al., 1997] to >40% [Landry et al., 2016]). In fact, few studies systematically addressed the IVEP outcomes throughout the prepubertal period. Landry et al. (2016), for example, retrospectively evaluated data from IVEP outcomes of Holstein donors aged 5, 6, 7, 8, 9, 10, 11, 12, and 16-18 mo, and observed a lower developmental competence of oocytes from those up to 10 mo, compared with pubertal heifers. This result was similar to the one reported by Presicce et al. (1997), who found lower developmental rates of oocytes retrieved from heifers up to 11 mo. Other studies, however, observed blastocyst rates in heifers from 6-9 mo similar to those of pubertal cattle (23.3% vs. 25.9%, respectively, Bernal-Ulloa et al., 2016). Among the difficulties to carry out comprehensive studies to investigate the effects of age on oocyte competence is the naturally high coefficient of variation of the antral follicle population in cattle, which is also observed in prepubertal heifers (Baldassarre et al., 2018; Feres et al., 2024). On the other hand, repeated follicle aspiration reduces oocyte yield over time, particularly in donors with greater antral follicle count (AFC) (Gimenes et al., 2015), which limits the control of individual variation by using the same calves as donors for a long period. Nevertheless, a systematic review of the literature (Tables 1 and 2) demonstrates that, regardless differences in methodology, blastocyst rates were consistently lower in prepubertal donors aged 7 mo or less (about 38% of the value of the control groups), whereas in older heifers the difference from the control was lower (about 67%) and frequently non-significant. This trend was observed for both *Bos taurus* and *Bos indicus*, in spite of the known difference in the age at puberty between these genetic groups (Nogueira 2004).

**Table 1:** Compilation of studies with *in vitro* embryo production using calf oocytes from *Bos taurus* breeds.

**Breed**	**Age (mo)**	**COC retrieval**	**Pre-** **treatment**	**BL rate** **(%)** **1**	**Control** **(%)** **2**	**P-value**	**Ratio** **(%)** **3**	**References**
Holstein	2-3	Laparot.	P4+FSH	11.0	--	--	--	Taneja et al. (2000)
Holstein	2-3.3	LOPU	P4+FSH	8.9	--	--	--	Baldassarre et al. (2018)
Holstein	2-3.3	LOPU	P4+FSH	12.8	--	--	--	Currin et al. (2017)
Holstein	3	Abattoir	No	8.7	20.6	<0.05	42.2	Revel et al. (1995)
Holstein	3	Abattoir	P4+FSH	10.5	27.0	<0.05	38.9	Revel et al. (1995)
Simmental	3	Abattoir	No	18.9	32.3	<0.05	58.5	Palma et al. (2001)
Unreported	2-4	Abattoir	No	11.4	30.1	<0.05	37.9	Kauffold et al. (2005)
Holstein	3.3-4.3	LOPU	P4+FSH	17.1	--	--	--	Currin et al. (2017)
Holstein	2.5-5.8	Laparot.	P4+FSH	6.0	40.0	<0.05	15.0	Damiani et al. (1996)
Holstein	4-5	Laparot.	P4+FSH	10.0	--	--	--	Taneja et al. (2000)
Holstein	5	OPU	No/FSH	0.0	18.0	<0.05	0.0	Presicce et al. (1997)
Holstein	5	OPU	FSH	31.4	48.0	<0.05	65.4	Landry et al. (2016)
Holstein	4.3-6	LOPU	P4+FSH	19.9	--	--	--	Baldassarre et al. (2018)
Holstein	4.3-6	LOPU	P4+FSH	21.8	--	--	--	Currin et al. (2017)
Crossbred	4-7	Abattoir	No	22.5	41.3	<0.05	54.5	Camargo et al. (2005)
Holstein	6	OPU	FSH	35.6	48.0	<0.05	74.2	Landry et al. (2016)
Holstein	6-7	OPU	FSH	1.0	24.0	<0.05	4.2	Oropeza et al. (2004)
Holstein	7	OPU	No	1.0	27.0	<0.05	3.7	Presicce et al. (1997)
Holstein	7	OPU	FSH	17.0	27.0	<0.05	63.0	Presicce et al. (1997)
Holstein	7	OPU	FSH	33.9	48.0	<0.05	70.6	Landry et al. (2016)
Holstein	6-9	OPU	No	23.3	25.9	ns	90.0	Bernal-Ulloa et al. (2016
Holstein	6-10	OPU	No	17.1	20.4	ns	83.8	Gutiérrez-Anez et al. (2021
Holstein	8	OPU	FSH	37.9	48.0	<0.05	79.0	Landry et al. (2016
Holstein	6.7-9.7	OPU	No	16.9	24.8	ns	68.1	Majerus et al. (1999)
Holstein	8.1-8.9	OPU	No	22.6	30.0	ns	75.3	Majerus et al. (1999)
Crossbred	9	Abattoir	No	9.4	--	--	--	Córdova et al. (2011)
Holstein	9	OPU	No	7.0	29.0	<0.05	24.1	Presicce et al. (1997)
Holstein	9	OPU	FSH	15.0	29.0	<0.05	51.7	Presicce et al. (1997)
Holstein	9	OPU	FSH	32.0	48.0	<0.05	66.7	Landry et al. (2016)
Holstein	9-10	OPU	FSH	9.0	24.0	ns	37.5	Oropeza et al. (2004)
Holstein	10	OPU	FSH	33.5	48.0	<0.05	69.8	Landry et al. (2016)
Holstein	<12	OPU	No	14.4	29.2	<0.05	49.3	Traut et al. (2022)
Holstein	11	OPU	No	24.0	41.0	<0.05	58.5	Presicce et al. (1997)
Holstein	11	OPU	FSH	23.0	41.0	<0.05	56.1	Presicce et al. (1997)
Holstein	11	OPU	FSH	42.0	48.0	ns	87.5	Landry et al. (2016)
Holstein	11-12	OPU	FSH	10.0	28.0	ns	35.7	Oropeza et al. (2004)
Crossbred	9-14	Abattoir	No	23.8	32.4	ns	73.5	Camargo et al. (2005)
Holstein	12	OPU	FSH	41.2	48.0	ns	85.8	Landry et al. (2016)

^1^ Blastocyst rates. In studies enrolling other treatments, we considered only data from the calf control group; ^2^ Blastocyst rate from the control group (pubertal heifers, cows, or abattoir), when present; ^3^ Ratio between the blastocyst rate from calves and from control [(BL rate calves/ BL rate control)*100].

**Table 2:** Compilation of studies with *in vitro* embryo production using calf oocytes from *Bos indicus* breeds.

**Breed**	**Age (mo)**	**COC retrieval**	**Pre-treatment**	**BL rate (%)** **1**	**Control (%)** **2**	**P-value**	**Ratio (%)** **3**	**Reference**
Nelore	3-4	LOPU	No	12.9	30.9	<0.05	41.7	Batista et al. (2016)
Nelore	3-4	LOPU	P4+FSH	11.3	30.9	<0.05	36.6	Batista et al. (2016)
Nelore	2-5	LOPU	No	31	71.6	<0.05	43.3	Kawamoto et al. (2022)
Nelore	4-7	OPU	No	1.3	24.4	<0.05	5.3	Zacarias et al. (2018)
Nelore	4-7	OPU	FSH	8.6	24.4	<0.05	35.2	Zacarias et al. (2018)
Nelore	6.5	OPU	No	24.4	36.3	<0.05	67.2	Toledo et al. (2023)
Nelore	8	OPU	No	35.4	54.5	<0.05	65.0	Moura (2022)
Nelore	8	OPU	FSH	41.4	54.5	ns	76.0	Moura (2022)
Nelore	8-11	OPU	No	42	48.1	ns	87.3	Kawamoto et al. (2022)
Nelore	8-12	OPU	No	20.2	47	<0.05	43.0	Baruselli et al. (2016)
Nelore	12	OPU	No	19.2	30.3	<0.05	63.4	Silva (2020)
Nelore	12	OPU	FSH	22.8	29.5	<0.05	77.3	Silva (2020
Nelore	12.7	OPU	No	16.4	--	--	--	Moura et al. (2023)
Nelore	12.7	OPU	FSH	31.6	--	--	--	Moura et al. (2023)
Nelore	13	OPU	No	27.1	28.3	ns	95.8	Silva et al. (2022)
Nelore	25	OPU	No	27.1	35.4	ns	76.6	Silva et al. (2022)
Nelore	18-22	OPU	No	28.1	47	<0.05	59.8	Baruselli et al. (2016)

^1^ Blastocyst rates. In studies enrolling other treatments, we considered only data from the calf control group; ^2^ Blastocyst rate from the control group (pubertal heifers, cows, or abattoir), when present; ^3^ Ratio between the blastocyst rate from calves and from control [(BL rate calves/ BL rate control)*100].

The progressive acquisition of *in vitro* developmental competence by the oocytes throughout the prepubertal period is associated with the morphological and functional development of the reproductive system, and indirectly with the somatic development. The ovarian and uterine size, the AFC, the number of large follicles, and the maximum size of the dominant follicle increase according to age (Rawlings et al., 2003; Nogueira, 2004; Kawamoto et al., 2022; Silva et al., 2022). Such changes, however, are neither linear nor do they reflect a progressive increase in circulating gonadotrophin concentrations.

**Figure 1:**
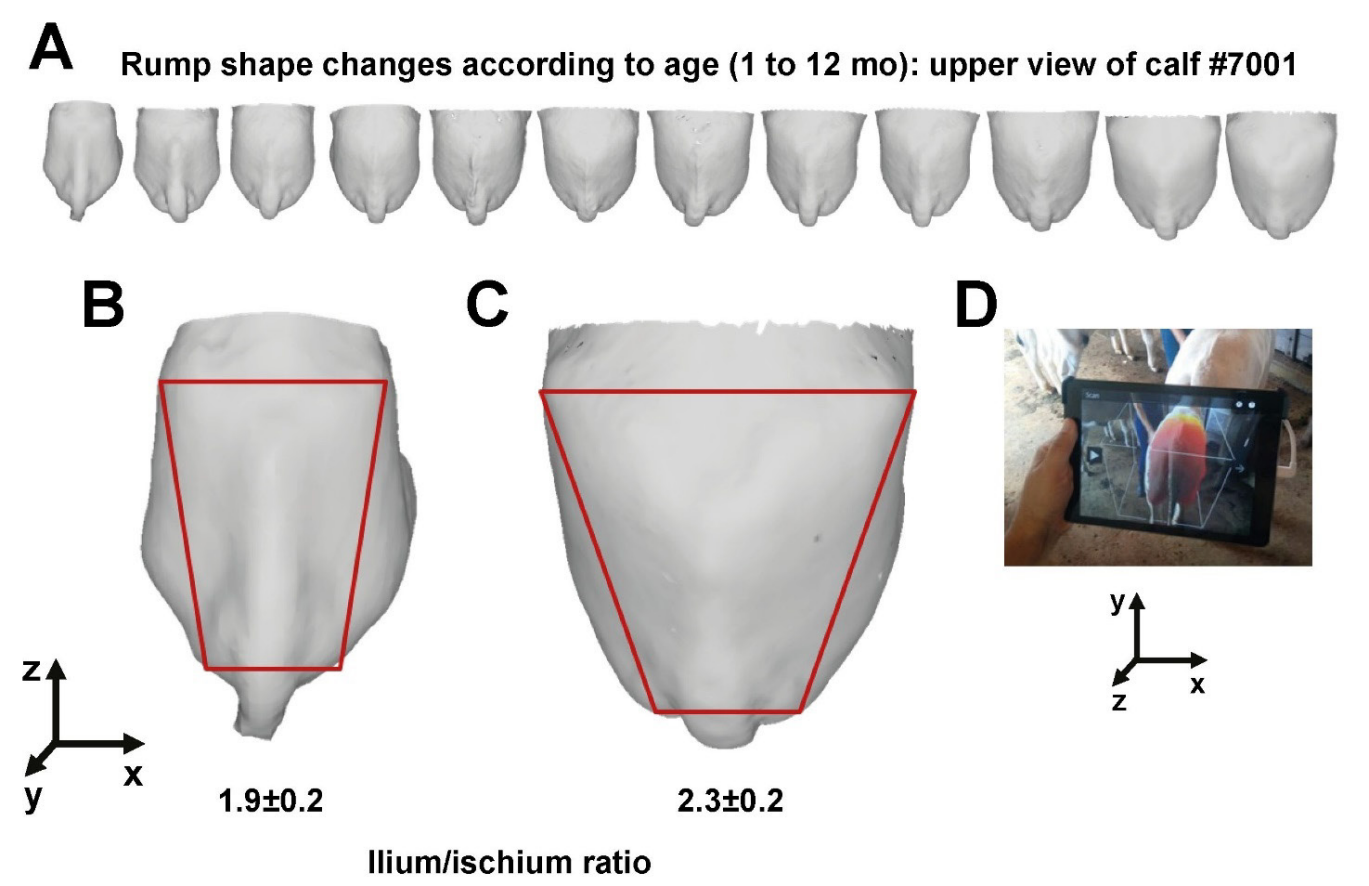
(A-D) Changes in rump geometry according to age. A) Upper view of 3D images from the rump area of a Nelore calf, obtained from one to 12 months-old (mo). The geometry differences from one (B) to 12 mo (C) is highlighted in red, with the corners of the trapezium corresponding to the lateral prominences of the ilium and ischium. D) Rear view of the calf, during a 3D scanning.

In the early prepubertal period, which comprises the first three to four months after birth, the hypothalamic-pituitary-gonadal axis undergoes a transitory activation, resulting in FSH and LH concentrations similar to those observed in adult cattle, both in *Bos taurus* (Evans et al., 1994) and *Bos indicus* (Kawamoto et al., 2022). This phenomenon is also observed in humans, being referred to as “minipuberty” (Kuiri-Hänninen et al., 2014), and results in ovarian follicle growth and estradiol (E2) production. Calves aged 2-4 mo from both Holstein (*Bos taurus*) and Nelore (*Bos indicus*) breeds presents anti-Müllerian hormone (AMH) concentrations greater than cycling heifers (Batista et al., 2016), reflecting the great number of preantral and early antral follicles developing in the ovaries. Interestingly, however, we also find during this period a remarkably low oocyte developmental competence *in vitro*, which is associated with a number of morphological and molecular features, including lower oocyte diameter (Kawamoto et al., 2022), cytoplasm biochemical composition (Salamone et al., 2001), delayed organelle redistribution following maturation (Damiani et al., 1996), abnormal chromatin configuration (Damiani et al., 1996), and altered gene expression patterns (Michalovic et al., 2018; Kawamoto et al., 2022; Silva et al., 2022). The physiological background for this apparent uncoupling between follicular growth and oocyte competence is not fully understood yet. One could speculate that ovarian steroids produced during this “minipuberty” play an important role in the development of the genital tract and secondary sexual characteristics, such as rump geometry (Figure 1). In a study with Nelore heifers in the early (3-5 mo) or intermediate (8-11 mo) prepubertal period, our group observed that the rate of development of a number of sexual characteristics was greater in the former than in the latter period, highlighting the importance of ovarian activity in young calves (Kawamoto et al., 2022).

The transition from the early to the intermediate prepubertal periods is characterized by an increase in the sensitivity of the hypothalamus to the negative feedback of ovarian E2, resulting in depressed LH concentrations (Evans et al., 1994; Nogueira, 2004). Therefore, although follicles can develop to greater sizes and the oocyte achieves a diameter and in developmental competence *in vitro* closer to those found in pubertal cows (Table 3), the final stages of follicle maturation and ovulation will not take place. The association between the progress in follicle development and oocyte developmental potential was previously demonstrated by Kauffold et al. (2005), who observed that blastocyst rates obtained with oocytes collected in unstimulated calves from large (>8 mm), but not from medium (4-8 mm) or small (2-3 mm) follicles, were similar to those from pubertal cattle.

**Table 3:** Proportion (%) of the values of reproductive endpoints measured during the early and intermediate prepubertal periods, in comparison with those observed in pubertal (set as 100%) cows (Adapted from Kawamoto et al., 2022).

**Endpoint**	**Prepubertal**	**Period**
	**Early (2 to 5 mo)**	**Intermediate (8 to 11 mo)**
Ovarian diameter	65.7%*	75.1%*
Antral Follicle Count (AFC)	73.1%*	83.3%*
Largest follicle diameter	71.3%*	82.3%*
Uterine diameter	65.6%*	80.8%*
Oocyte diameter	96.3%*	99.0%
Blastocyst rate after IVF	43.8%*	78.8%
Plasma FSH concentration	106.6%	77.5%*

* Indicates statistical difference (P<0.05) from the reference value (adult cows).

The somatic development eventually causes a progressive reduction in the hypothalamic sensitivity to E2, and consequently an increase in LH pulse frequency and amplitude that will ultimately lead to the first ovulation and to the beginning of estrous cycles, i.e., puberty. Therefore, the acquisition of maturity by the hypothalamic-pituitary-ovarian axis with be also affected by factors such as nutrition and genetic background and, indirectly, season (Nogueira, 2004; Ferraz et al., 2018). Puberty generally occurs shortly after the intermediate period in *Bos taurus* breeds, with many animals beginning estrous cycles before 12 mo (Rawlings et al., 2003). Peripubertal Holstein heifers with 10-13 mo, for example, can be successfully induced to superovulation and be flushed to collect embryos *in vivo*, without any detrimental effects on further fertility (Fernandes et al., 2020).

*Bos indicus* breeds, on the other hand, are known to be less precocious. Zebu breeds have an average age at puberty around 22-36 months, despite the increase in LH pulse frequency and the associated increase in maximum size of the dominant follicles that takes place after 12 mo (Nogueira, 2004). There is, however, a significant variation in the age at puberty in *Bos indicus*, and precocious heifers can become pregnant before 15 mo. In fact, Ferraz et al. (2018) demonstrated that Nelore heifers born from sires with low expected progeny difference for age at first calving and raised to have a high average daily gain (0.7 kg) achieved puberty on average at 18.1 mo. In commercial IVEP routines, there is a natural bias towards the use of such heifers with high genetic merit, including for age at first calving, and that receive diets with high levels of total digestible nutrients. Moreover, prepubertal heifers frequently undergo hormonal protocols consisting of steroids and gonadotrophic stimulation before ovum pick-up (OPU), which precludes the correct identification of the individual age at puberty. Therefore, the arbitrary categorization of *Bos indicus* heifers aged between 12 and 24 months and used as oocyte donors as “prepubertal” may actually be imprecise, so we opted to call this period the “late prepubertal” or “peripubertal” period.

**Figure 2:**
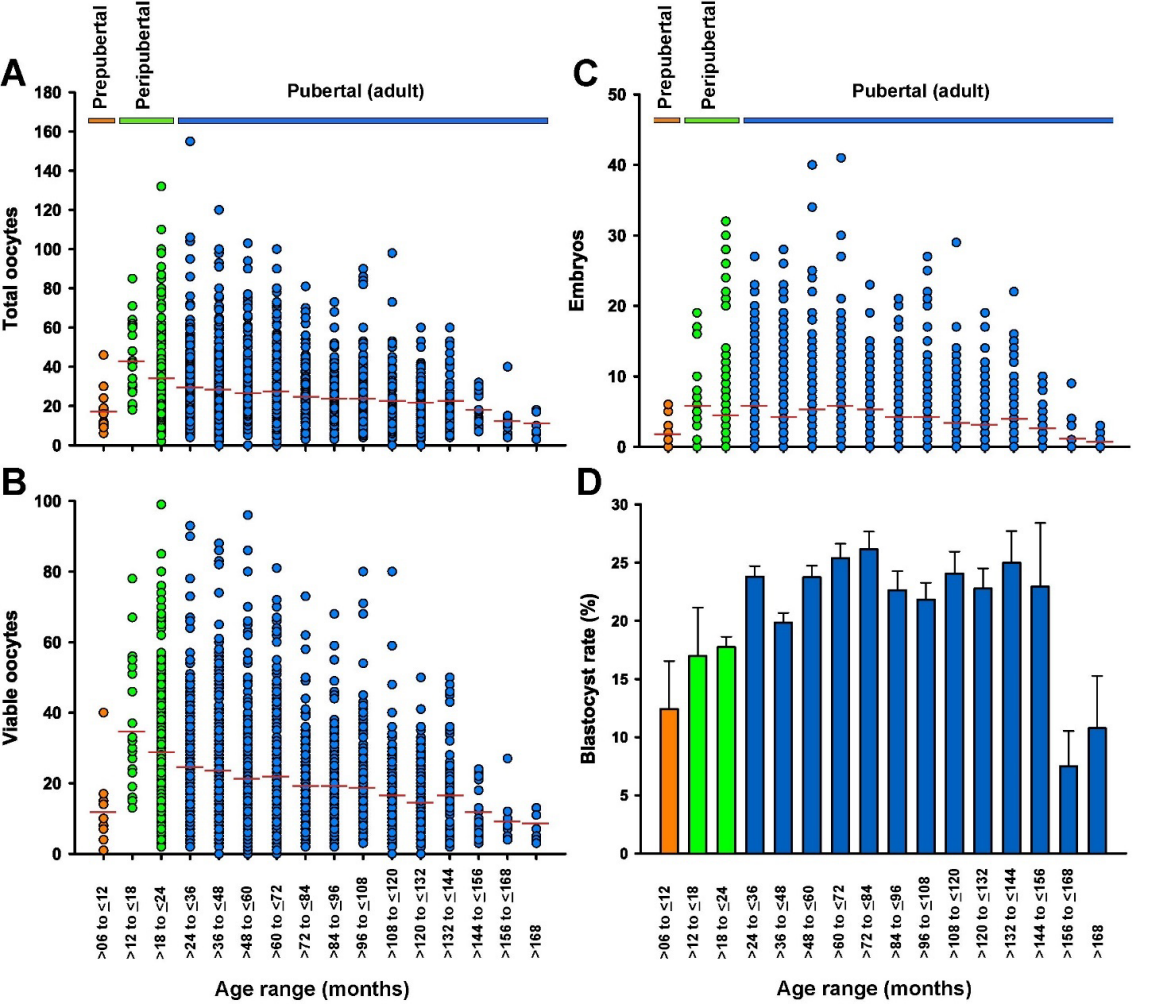
(A-D) Data from OPU-IVEP outcomes of 3,030 Gir breed donors collected between 2018 and 2020, stratified according to age range (months). A-C) The dots represent the values of individual donors, and the red lines the average for each endpoint and age range. Heifers from 6 to 12 months old were considered as prepubertal (orange dots), whereas those from 12,1 to 24 months old were classified as peripubertal (green dots). D) Blastocyst rates (mean±SEM), according to age range.

The late puberty in *Bos indicus* cattle is also reflected in the developmental potential of the oocytes recovered. Figure 2 depicts the results of 3,030 OPU-IVEP sessions performed in Gir breed donors, from the same farm, stratified according to age ranges (in months) (Feres, personal communication). In spite of the predictable individual variation, the average oocyte and embryo yield (Fig. 2 A-C) increased from the prepubertal (6-12 mo) to the peripubertal period (12.1-24 mo), but not afterwards. In fact, the number of viable oocytes recovered and embryos produced from prepubertal heifers were lower than those observed in peripubertal and in pubertal donors aged 2-12 years old, but similar to those from older (>12 years old) cows (12.3±2.8^c^ *vs.* 28.0±0.9^a^, 20.9±0.3^b^, and 10.4±0.5^c^ oocytes, and 1.8±0.5^b^ *vs.* 4.4±0.3^a^, 4.6±0.1^a^, and 1.9±0.2^b^ embryos, respectively, P<0.05). The blastocyst rates (Fig. 2 D), however, were still lower in the peripubertal period than in adult cows (17.7±0.9% *vs.* 23.2±0.4%, respectively, P<0.0001). A similar trend was observed by Baruselli et al. (2016) when comparing peripubertal (18-22 mo) with pubertal (22-26 mo) heifers, with a greater number of follicles and oocytes retrieved in the former, but a greater blastocyst rate in the latter.

The increase in ovarian activity observed throughout the intermediate prepubertal and peripubertal periods is consistent with the changes in AMH concentrations. Guerreiro et al. (2014) observed that plasma AMH in prepubertal Nelore heifers increased from 0.7±0.1 ng/mL at 10-11 mo to 1.4±0.3 at 21-23 mo, but not thereafter (1.4±0.4 at 24-26). The increase in blastocyst rates during the peripubertal phase, however, is not proportional to those in the number of follicles and oocytes retrieved (Fig. 2D). This may potentially reduce the efficiency of AMH as an endocrine marker for the selection of calves and prepubertal heifers as oocyte donors for IVEP. Although heifers with greater AMH yield more oocytes and therefore more embryos, they tend to have lower blastocyst rates (Feres et al., 2024), so the correlation between AMH and blastocyst rate is low and not significant (Guerreiro et al., 2014).

Altogether, these data suggests that during the peripubertal period there is substantial increase in ovarian activity and in the number of follicles growing on the ovaries. The oocyte developmental potential, on the other hand, will only reach its maximum after puberty, even though this may occur many months later. Whether anticipating puberty in the Zebu, either by genetic selection or pharmacological induction, would allow an earlier increase in oocyte developmental potential remains to be evaluated.

### Gonadotrophic pre-stimulation before follicle aspiration

Ovarian pre-stimulation with FSH is the most common strategy to improve laparoscopic ovum pick-up (LOPU) or OPU outcomes, considering both oocyte number and *in vitro* developmental potential. Superstimulation increases the number of follicles that can be identified and punctured on the ovary surface, and thus has been used in most studies with LOPU in calves (Currin et al., 2021). In calves collected by OPU, the aspiration of large follicles results in a greater amount of follicular fluid, which reduces turbulence in the aspiration line and therefore contributes to preserving COC from being denuded. The pre-stimulation with FSH also has direct beneficial effects on the cumulus-oocyte complexes, such as increased mRNA of genes related to E2 synthesis and follicular growth (FSHR and CYP19A1) (Michalovic et al., 2018).

To what extent the use of exogenous FSH compensates for the intrinsic low developmental competence of prepubertal oocytes, however, has been a matter of debate. Differences in age, breed, and stimulation protocols may explain some of the inconsistencies among studies. For instance, Presicce et al. (1997) reported a beneficial effect of FSH priming in Holstein heifers from 7 to 9 mo, but not at 11 mo. This difference is consistent with the lower average size achieved by the follicles in younger heifers, and thus with the associated lower oocyte developmental potential (Kauffold et al., 2005; Kawamoto et al., 2022). On the other hand, as heifers reach the peripubertal period, differences from pubertal animals tend to become non-significant, and the use of FSH will take into account other factors, such as the cost of treatment.

The breed also seems to interact with the response to FSH in calves. In pubertal *Bos taurus* cattle, gonadotrophic pre-stimulation is known to improve embryo outcomes (Sarwar et al., 2020) and has been widely used before OPU. Conversely, FSH is seldom used in the commercial OPU routine in *Bos indicus* breeds (Viana, 2023). The lack of consensus on the benefits of FSH priming in the Zebu may be due to the greater importance of other sources of variation, such as the greater range in AFC (Feres et al., 2024) and sensitivity to gonadotrophins (Wohlres-Viana et al., 2019). Coherently, the effects of FSH priming before OPU in prepubertal calves and heifers seem more consistent in *Bos taurus* than in *Bos indicus*. Previous studies reported an increase in oocyte developmental potential after treatment of Holstein heifers with FSH (Revel et al., 1995; Presicce et al., 1997), even though blastocyst rates remained lower than those from adult cattle. On the other hand, Batista et al. (2016), Zacarias et al. (2018) and Silva (2020) found an increase in the number of follicles visualized and oocytes retrieved, but not in blastocyst rates, after using FSH priming in Nelore calves.

Most pre-stimulation protocols for *Bos taurus* prepubertal calves and heifers were based on porcine FSH (pFSH), with doses ranging from 60 to 240 mg Folltropin (Oropeza et al., 2004; Landry et al. 2016), and starting 36 to 72h before OPU. In this regard, Currin et al. (2017) demonstrated that long (72h), but not short (36h), stimulation protocols were effective in improving blastocyst rates in prepubertal cattle. The rationale for the use of long protocols is that follicles would achieve greater size and, therefore, support the acquisition of greater developmental potential by the oocytes (Kauffold et al., 2005; Baldassarre et al., 2018). An alternative to avoid the multiple injections required by the pFSH in such longer protocols is the combined use of eCG (Baldassarre et al., 2018). To explore the hypothesis that increasing the length of the stimulation protocol would be beneficial for Zebu breeds, while preventing the stress associated with repeated injections, our group evaluated extended protocols (96 or 120h) before OPU in Nelore prepubertal heifers, using a single injection of a long-acting recombinant human FSH (rhFSH) (Moura et al., 2023). In both extended protocols, rhFSH was effective to induce superstimulation (100% and 92.3%, respectively, *vs.* 13.3% using eCG and 0.0% in untreated controls, P<0.0001) and to increase COC quality (52.4% and 37.0% of grade I COC, *vs.* 15.3% with eCG and 16.4% in untreated controls, P<0.0001). Moreover, when OPU was carried out 120h after rhFSH, blastocyst rates were similar to those obtained from adult cows (41.4% *vs.* 54.;5%, P>0.05). However, the greater average follicle size was associated with a lower recovery rate, resulting in a similar number of blastocysts produced per OPU (2.3±0.4 vs 2.8±0.7% in untreated controls, P<0.0001). This lower recovery rate was also observed, though to a lesser magnitude, even with shorter (48h) protocols (Batista et al., 2016), which failed to improve IVEP outcomes. These results suggest that longer stimulation protocols might be an alternative to improve embryo yield in Zebu prepubertal heifers, as long as the aspiration procedures are optimized for large follicles.

#### Impacts on further fertility

The recovery of oocytes by LOPU or OPU may cause morphological and functional sequelae such as tunica albuginea thickening, ovarian fibrosis, and adherence (Petyim et al., 2001; Viana et al., 2003). Moreover, the successive removal of growing follicles may induce endocrine imbalances (Viana et al., 2010), and result in the development of chronic cystic ovarian disease (COD, Viana et al., 2021). In adult cattle, however, the side effects of OPU have been largely neglected. A possible explanation for this low concern with donor reproductive health is the large number of offspring that can be generated by IVEP in a relatively short period of time. Therefore, the rapid turnover of donors would make such individual loss of fertility less relevant for the breeding programs. The scenario is different when it comes to younger animals. Although there is a lack of comprehensive studies on the consequences of LOPU or OPU on young cattle, field reports, anatomical features, and other indirect evidence suggest that calves could be more prone to develop ovarian sequelae. Therefore, their potential as oocyte donors could be compromised before their genetic potential is fully explored.

The somatic development is the major limitation for the possible approaches to obtain embryos from calves, as during the early prepubertal period (up to 4-5 mo) transrectal palpation is not feasible and the genital tract can only be accessed by laparoscopy or surgery. The LOPU can be carried out in calves as early as two mo (Currin et al., 2017; Baldassarre et al., 2018), and has been the technique of choice to retrieve oocyte during this period. On the other hand, from approximately six mo onwards (depending on the breed) ovaries can be manipulated by rectal palpation, and OPU can be carried out with vaginal probes and needle guides of small diameter, usually adapted from those used in human medicine (Presicce et al., 1997; Majerus et al., 1999). Although weight is generally used as the main index of body development to select heifers for OPU, the size of the pelvis and perineal region is also important. In an experiment with 6.3-7.7 mo Nelore calves, rectal palpation could not be carried out in two out of 10 animals, but both were above the average weight of the group (214.0 and 206.0 kg vs 205.6±4.0 kg, personal data).

**Figure 3:**
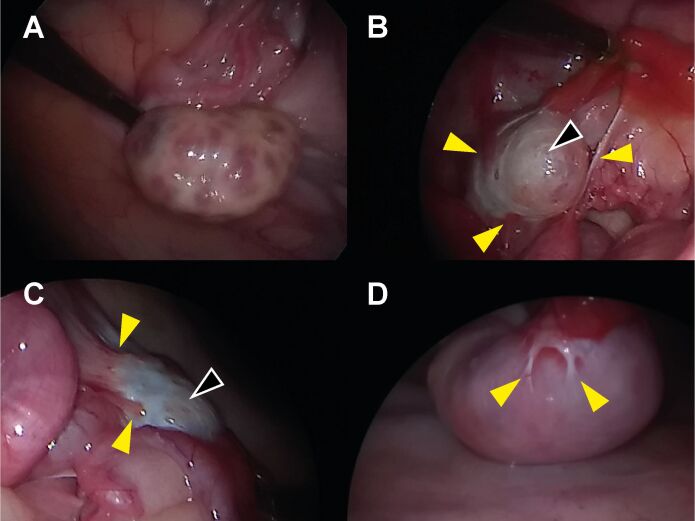
(A-D) Laparoscopic images of the genital tract Gir heifers that were (B-D) or were not (A) subjected to oocyte retrieval by LOPU and OPU in the prepubertal period. A) Control heifer. The ovary is free of adhesions and antral follicles can be easily observed in its surface. B-D) Sequels of LOPU/OPU. Tunica albuginea thickening is indicating by black arrows. Adhesion points in the ovaries (B, C) and uterus (D) are indicated by yellow arrows.

The LOPU approach has the advantage of allowing direct visualization of the follicles to be aspirated but requires three abdominal incisions, respectively for the optics, forceps, and aspiration needle guide, which is a risk factor for the development of adhesions. The transvaginal approach (OPU) is a needle biopsy and, thus, considered to be less invasive, besides avoiding fasting and sedation. Calves’ ovaries, however, are smaller and may be more difficult to handle properly, particularly considering that most farms do not have squeeze shuttles adapted for small, prepubertal cattle. Moreover, the FSH priming, frequently done to prepare calves for LOPU or OPU, increases ovarian vascularization (Abdelnaby et al., 2023), and therefore may also increase subsequent bleeding, another risk factor for the development of adhesions. Our group carried out laparoscopic evaluations of heifers that underwent six sessions of LOPU during the early and six sessions of OPU during the intermediate prepubertal periods. The images revealed that most females presented ovarian sequelae such as tunica albuginea thickening and adhesions (Figure 3, personal data).

Finally, it remains to be studied whether or not the endocrine manipulation aiming at improving IVEP outcomes could impact further productive life of calves. Strategies such as the use of gonadotropins, bovine somatotropin (bST), or steroids before OPU cause an endocrine imbalance, which is transient but takes place in a critical window of the female somatic and reproductive development, and thus might potentially affect their subsequent productive performance.
